# Developing the Public Speaking Anxiety Scale (PSAS) for Adolescents: The Mediating Role of Dysfunctional Emotion Regulation in the Effect of Irrational Beliefs on Public Speaking Anxiety

**DOI:** 10.3390/bs15060825

**Published:** 2025-06-16

**Authors:** Sezai Demir, Mustafa Onur Kan

**Affiliations:** 1 Department of Guidance and Psychological Counselling, Faculty of Education, Hatay Mustafa Kemal University, Antakya 31060, Türkiye; sezaidemir@mku.edu.tr; 2 Department of Basic Education, Faculty of Education, Hatay Mustafa Kemal University, Antakya 31060, Türkiye

**Keywords:** public speaking anxiety, dysfunctional emotion regulation, irrational beliefs, adolescents

## Abstract

Public speaking anxiety, which is closely related to social anxiety, is a crucial factor in the development of adolescents. It affects their ability to regulate their emotions and irrational beliefs, which in turn shapes their relationships and academic success. The purpose of this present study is two-fold: (a) to develop a valid and reliable measurement tool for public speaking anxiety for adolescents, and (b) to determine the mediating role of dysfunctional emotion regulation on the effect of irrational beliefs on public speaking anxiety. To achieve this, data were collected through face-to-face interviews from a total of 1231 adolescent students, including 642 girls (age, $\bar{X}$ = 14.96) and 589 boys ($\bar{X}$ = 14.99), aged between 12 and 17 years old in five stages. Data collection was based on the Public Speaking Anxiety Scale (PSAS) (developed in the current study), the Irrational Beliefs Scale (IBS), the Regulation of Emotions Questionnaire (REQ) and the Social Anxiety Scale for Adolescents (SAS-A). Data were analysed through SPSS, AMOS, JAMOVI, G-POWER and Microsoft Excel programmes. This study concludes that the Public Speaking Anxiety Scale (PSAS) has demonstrated both valid and reliable psychometric properties. The findings of this study further reveal that internal dysfunctional emotion regulation plays a partial mediating role in the effect of irrational beliefs on public speaking anxiety, and that external dysfunctional emotion regulation, on the contrary, did not have a mediating role in the effect of irrational beliefs on public speaking anxiety.

## 1. Introduction

The adolescent period is characterised by a series of challenging life events (Nicolson & Ayers, 2004; Santrock, 1987), which have a significant impact on the individual’s developmental process (Dacey & Kenny, 1997; Steinberg & Morris, 2001). Given that adolescents are highly sensitive to their environment (Smetana et al., 2006), social anxiety represents a significant challenge during this developmental period. This is particularly prevalent between the ages of 11 and 17 (APA, 2013). It has been established that public speaking anxiety is a prevalent phenomenon among individuals with social anxiety disorder, with an estimated prevalence rate of approximately 97% (Beidel & Turner, 2007). However, this rate also demonstrates a significant positive correlation with social anxiety, indicating a high degree of comorbidity between these two conditions (Fremouw & Breitenstein, 1990; Harb et al., 2003; Miers et al., 2014). It is widely acknowledged that public speaking anxiety represents the most prevalent form of social anxiety disorder, with a documented prevalence rate of 7% over the course of a year (APA, 2013).

Speaking anxiety is a sub-dimension of social anxiety (Fremouw & Breitenstein, 1990; Harb et al., 2003; Miers et al., 2014) that is associated with a fear of negative evaluation (Breakey, 2005; Feldman & Silvia, 2010). It is manifested through emotional reactions such as fear, sadness and anger, physical reactions such as a rapid heartbeat and sweating, and behavioural reactions such as avoidance. This anxious reaction to speaking can be observed in a variety of settings, including spontaneous situations in daily life, in prepared speech in front of an audience (Pertaub et al., 2002), in classroom activities, during shopping, and in dialogue with familiar individuals. Speaking anxiety has been demonstrated to affect individuals at every stage of developing their speaking skills (Behnke & Beatty, 1981; Stein et al., 1996). Furthermore, an increase in anxiety levels has been shown to result in an avoidance of speaking or a reaction (Behnke & Beatty, 1981; Stein et al., 1996). The core issue under consideration is the prevalence of negative beliefs concerning the prospect of unfavourable evaluation. These beliefs give rise to a subsequent reaction, as evidenced by previous studies (Behnke & Beatty, 1981). When considering the evaluation of speaking anxiety, with regard to the individual’s cognitive features, irrational beliefs assume particular significance.

Irrational beliefs are recognised as a concept belonging to the cognitive behavioural approach. This approach establishes a cause-and-effect relationship that underlies numerous problems, ranging from fundamental communication difficulties to mental health concerns (David et al., 2009). Irrational beliefs, also known as cognitive distortions, consist of three dimensions: the demandingness of the individual to themself, to others and to the environment/world in which they live (David et al., 2009; Ellis, 2013). Irrational beliefs, which are based on false ideas and have little or no confirmatory dimension, are also observed in adolescents (Leigh & Clark, 2016). The repercussions of perfectionism in adolescence may encompass elevated stress levels (Yıldız et al., 2018), the manifestation of anger and aggressive behaviours (Fives et al., 2011), diminished life satisfaction (Çivitçi, 2009), and the development of perfectionist personality traits (Flett et al., 2008). According to Ellis (2013), the experience of dysfunctional emotions, which are the result of irrational beliefs, is thought to cause adolescents to experience their emotions inappropriately and to experience problems in emotion dysregulation processes. Irrational beliefs in adolescents have been shown to cause problems in emotion dysregulation processes and to result in dysfunctional emotions (Ellis, 2013). In this regard, the regulation of adolescents’ dysfunctional emotions assumes particular significance.

Emotion regulation is a pivotal process for adolescents. This is due to the fact that they experience their emotions differently from adults and children (Gross & John, 2003), because they are exposed to rapid physical and biological changes, and their self-development (Steinberg & Morris, 2001). According to Thompson (1994), emotion regulation can be defined as the suite of cognitive processes developed by the individual to monitor, evaluate and improve their temporary and intense emotions in order to achieve their goals. Emotion regulation is defined as a process that involves the intentional reduction or maintenance of desired emotional responses. This process is facilitated by either deliberate effort or spontaneity, through internal and external mechanisms. The term “internal dysfunctional emotion regulation” is used to denote the inability of the individual to regulate their emotions and to reflect negatively on themselves. It is possible to provide examples of regulatory processes that may be observed in such cases as suppression, rumination and self-blame. The concept of external dysfunctional emotion regulation can be defined as the experience of uncontrollable emotions, with the subsequent direction of their consequences or causes to external sources. Examples of this situation include aggressive behaviour, blaming others, avoidance, and mistreating vulnerable people (Ambrosini et al., 2013; Gratz & Roemer, 2004; Philips & Power, 2007). Furthermore, an additional illustration of internal dysfunctional emotion regulation is characterised by symptoms such as over-arousal and mental instability. Conversely, external dysfunctional emotion regulation is typified by the occurrence of tantrums and the infliction of harm upon others (Paulus et al., 2021). Conversely, dysfunctional emotion regulation has been a subject of considerable research interest in recent years (Cécillon et al., 2024; Gratz et al., 2017; Hatkevich et al., 2021; Vacca et al., 2023), due to its deleterious effects on emotional development, cognitive and behavioural adaptation, self-efficacy, social relationships and functionality, and quality of life.

### Present Study

Adolescents may experience performance-related speaking anxiety (Blöte et al., 2014; Chiu et al., 2021), which is a subcomponent of social anxiety (Leigh & Clark, 2016), due to the concern that they will be ridiculed. It has been established that this anxiety experienced by adolescents in front of the public has a detrimental effect on their interpersonal relationships (Urbán et al., 2024), and that it can also hinder their ability to demonstrate their talents and achievements (McNatt, 2019). As posited by Behnke et al. (1987), there is a paucity of correlation between subjective reports and observations in the measurement of speaking anxiety. Furthermore, Mulac and Sherman (1974) and Sypher (1980) posit that non-test techniques make biased measurements and ignore personal differences (Monson & Snyder, 1977). In addition, Gallego et al. (2022) utilised a combination of self-report, audience and physiological observation techniques to assess public speaking anxiety, concluding that self-report emerged as the most valid method. In this direction, it has been observed that Likert-type measurement tools for public speaking anxiety, which have been found to be effective in adolescents’ relationships and skills, have been developed or adapted exclusively by language educators (Çabuker et al., 2020; Demir & Melanlıoğlu, 2014; Gündüz & Demir, 2021; Keşaplı & Çifçi, 2019; Kinay & Özkan, 2014; Sevim, 2012; Yaman & Sofu, 2014). It is understood that measurement tools developed outside Turkey are exclusively for university students (Bartholomay & Houlihan, 2016; Gallego et al., 2022; Hofmann & DiBartolo, 2000; Lin et al., 2021; Mörtberg et al., 2018). While the university period is recognised as being pivotal in terms of future preparation and career development, it is acknowledged that the adolescence period (Santrock, 1987; Viner et al., 2015) plays a more critical role in terms of personality development and future readiness. Furthermore, APA (2013) stated that the most common age range of social anxiety, which includes public speaking anxiety, is 11–17 years old. In this context, the importance of a measurement tool to determine the effects of speaking anxiety, which is seen to be important for adolescence, has become increasingly evident.

As evidenced by previous research, dysfunctional emotion regulation strategies have been demonstrated to be a contributing factor to a multitude of psychiatric disorders and life problems experienced by adolescents (Paulus et al., 2021; Schweizer et al., 2020; Young et al., 2019). These strategies have been shown to have a mediating role in this particular study. Social anxiety associated with public speaking anxiety has been shown to be caused by negative emotional regulation (Bates et al., 2021; Carthy et al., 2010; Golombek et al., 2020) and cognitive errors (Brandrick et al., 2021; Clark & Wells, 1995; Muris & Field, 2008). David et al. (2002) propose that functional and dysfunctional emotion regulation have two distinct origins. The initial proposition is that, for emotion regulation to be effective, the quality rather than the intensity is paramount. Secondly, irrational beliefs are identified as a contributing factor to dysfunctional emotion regulation. Individuals harbouring irrational beliefs concerning the regulation of their emotions often encounter difficulties in achieving effective emotional regulation (Castella et al., 2018). Consequently, they have a tendency to avoid their experiences cognitively and behaviourally, based on negative emotional outcomes (Brockmeyer et al., 2012). As Trincas et al. (2016) have previously asserted, the intensive utilisation of cognitive strategies, such as rumination, which is associated with irrational beliefs, is effective in cases of an inability to regulate emotions in a functional manner. Adolescents who demonstrate capacity for rational thought have been found to experience reduced anxiety (Choi & Kim, 2008; Moshman, 2013) through the regulation of their emotions (Rice et al., 2017; Roiser et al., 2012; Rutter et al., 2001). It has been established that there is a relationship between trait anxiety, metacognitive skills, and emotion dysregulation, which is analogous to public speaking anxiety (Cécillon et al., 2024). Adolescents who harbour the irrational belief that they will be the subject of ridicule (Blöte et al., 2014; Chiu et al., 2021; Leigh & Clark, 2016; Miers et al., 2014) have been shown to experience elevated levels of anxiety in situations that demand performance (Chiu et al., 2021; Leigh & Clark, 2016). Moloudi et al. (2022) also demonstrated that, in their CBT-based experimental study for high school students, social anxiety decreased as irrational beliefs decreased. Conversely, irrational beliefs have been demonstrated to have a substantial capacity to predict social anxiety (Davison & Zighelboim, 1987; Şahan & Kahtali, 2021; Tovilović, 2004) and speaking anxiety (Lohr & Bonge, 1981; Şahan & Kahtali, 2021). In addition to these direct relationships, Fremouw and Zitter (1978) proposed that other factors should also be considered in the relationship between irrational beliefs and public speaking anxiety. Ellis (2013) hypothesised that irrational beliefs are a contributing factor to unregulated negative emotions, which in turn can lead to anxiety. Furthermore, Leigh and Clark’s (2016) seminal study posited that individuals with irrational beliefs exhibit diminished levels of social anxiety, a phenomenon attributable, at least in part, to their adeptness at emotion regulation.

It is therefore evident that the level of anxiety experienced in activities such as public speaking, which necessitate public performance through the regulation of irrational beliefs during adolescence, affects both academic development (De Lijster et al., 2018; Fisher et al., 2004) and social development (Biggs et al., 2012; Blöte et al., 2014). It is postulated that the development of a measurement instrument for the assessment of public speaking anxiety among adolescents, accompanied by the elucidation of the intercorrelations among its putative determinants, will contribute to closing the gap in the field at the preliminary level. The present study is a pioneering interdisciplinary investigation into speaking anxiety from the perspectives of linguistics and psychological counselling. It is anticipated that the development of the first public speaking anxiety scale for adolescents and the investigation of the impact of irrational beliefs and emotion dysregulation on public speaking anxiety will provide valuable insights that can inform the work of school guidance services.

The objective was to develop a measurement tool for public speaking anxiety in high school students and to examine the mediating role of emotion dysregulation in the effect of irrational beliefs on public speaking anxiety. To achieve this general aim, this study was conducted in two stages.

Study 1: Determining the psychometric properties of the Public Speaking Anxiety Scale.

Study 2: Examining the mediating role of internal and external dysfunctional emotion regulation on the effect of irrational beliefs on public speaking anxiety in adolescents.

**H_1_.**  *The overall effect of irrational beliefs on public speaking anxiety in adolescent students is positively significant.*

**H_2_.**  *The direct effect of irrational beliefs on internal dysfunctional emotion regulation in adolescent students is positively significant.*

**H_3_.**  *The direct effect of irrational beliefs on external dysfunctional emotion regulation in adolescent students is positively significant.*

**H_4_.**  *The direct effect of internal dysfunctional emotion regulation on irrational beliefs in adolescent students is positively significant.*

**H_5_.**  *The direct effect of external dysfunctional emotion regulation on irrational beliefs in adolescent students is positively significant.*

**H_6_.**  *There is a significant positive relationship between internal dysfunctional emotion regulation and external dysfunctional emotion regulation in adolescent students.*

**H_7_.**  *The indirect effect of irrational beliefs on public speaking anxiety through internal dysfunctional emotion regulation is positively significant in adolescent students.*

**H_8_.**  *The indirect effect of irrational beliefs on public speaking anxiety through external dysfunctional emotion regulation is positively significant in adolescent students.*

**Figure 1:**
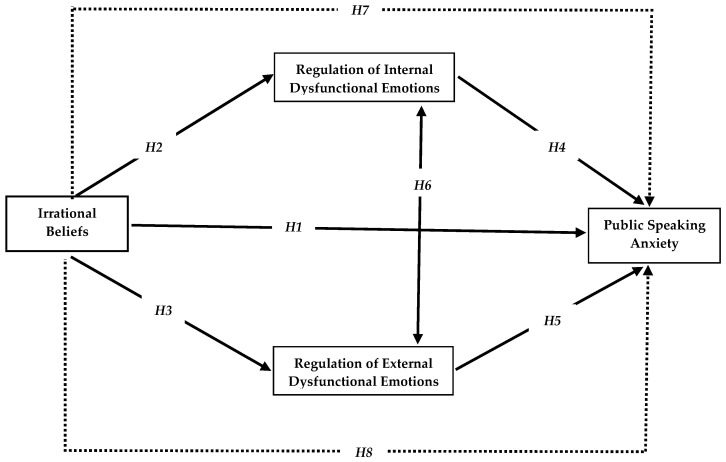
Hypotheses on the mediating role of internal dysfunctional and external dysfunctional emotion regulation in the effect of irrational beliefs on public speaking anxiety in adolescent students.

The hypotheses of the research are presented in Figure 1:

## 2. Methods

### 2.1. Research Model

This study employs a descriptive research design based on relational survey model. As the research process consists of two parts, psychometric studies are carried out in the first stage to develop a public speaking anxiety scale for adolescents. The second stage, using the developed Public Speaking Anxiety Scale, probes into the mediating role of internal dysfunctional and external dysfunctional emotion regulation in the effect of adolescents’ irrational beliefs on public speaking anxiety.

### 2.2. Study Group and Data Collection Process

This study has been conducted with the permission of the Social and Human Sciences Ethics Committee of Mersin University, dated 3 November 2023, and numbered 25. Convenience sampling was used in the research process to determine the level of relationships between variables, and data were collected face-to-face from a total of 1231 students, including 642 girls and 589 boys, pursuing their education in secondary schools and high schools in Adana, Sakarya, and Mersin provinces in Turkey. Permission was obtained from the families of the students through the parental consent form during the data collection process. The form in question was submitted to the relevant board, accompanied by the requisite ethical approval. The age of the sample group varies between 12 and 17 years old. The average age of the total sample in both stages is ($\bar{X}$ = 14.96) for girls and ($\bar{X}$ = 14.99) for boys. The research process involved five different sample groups in different stages. In the first stage, which included the development of a scale, 20 (11 girls, 9 boys; age $\bar{X}$ = 14.9) students participated in the study for focus group interviews in the first step, and 520 (272 girls, 248 boys; age $\bar{X}$ = 15.2) students for the exploratory factor analysis in the second stage. Also, 210 (109 girls, 101 boys; age $\bar{X}$ = 14.9) students took part in this study for the composite reliability (CR) analysis as well as confirmatory factor analysis in the third stage. For the fourth stage, 160 (82 girls, 78 boys; age $\bar{X}$ = 15.1) students participated in the validity test through the average variance extracted (AVE) analysis and in the reliability tests through the criterion scale, internal consistency and test retest method; for the final step in the second stage, 321 (168 girls, 153 boys; age $\bar{X}$ = 14.8) adolescent secondary and high-school students took part in the relational and mediation analyses.

### 2.3. Data Collection Tools

#### 2.3.1. Public Speaking Anxiety Scale (PSAS)

The development of the PSAS was informed by a comprehensive analysis of the extant literature pertaining to social and public speaking anxiety. It has previously been established that the reactions associated with such anxieties can manifest in physiological (sweating, blushing, hot and cold flushes, et cetera), behavioural (avoidance) and cognitive (thinking that they will be humiliated, believing that they will not succeed, etc.) forms (as cited in Behnke & Beatty, 1981; McCroskey, 1977, 1982; Lang, 1969). In this context, 39 items were obtained. Subsequently, a focus group interview was conducted with 20 adolescents (11 girls, 9 boys) aged between 11 and 17 years old on ‘their experiences during public speaking when they have to speak in front of the public’. The content analysis yielded 15 items. Following a thorough analysis and review of the relevant literature, a total of 54 items were obtained. In the creation of the items, particular attention was paid to the cognitive, physiological and avoidance reactions of public speaking anxiety, and the expressions of emotions, thoughts, and behaviours that should be included in Likert-type scales (DeVellis & Thorpe, 2021). The opinions of four faculty members from the field of Guidance and Psychological Counselling, two faculty members from the field of Measurement and Evaluation, and one faculty member from the field of Psychology were collated, and the number of items was determined as 29. In order to ascertain the linguistic compatibility of the items in question, the opinions of two faculty members, who are experts in the field of language education, were also taken into consideration. The validity and reliability results of this scale are presented in Section 3 under Study 1.

#### 2.3.2. Irrational Beliefs Scale (IBS)

This measurement tool was adapted to high school students by Türküm (2003) through shortening the Irrational Beliefs Scale designed by Türküm et al. (2005) for university students. This scale has 16 items and 1 dimension. Higher scores on this scale indicate higher irrational beliefs. This is a 5-point Likert-type scale, with no reversed items. The minimum score achievable from the scale is 16, while the maximum score is 80. The structural validity of the scale was tested, and the common variances of the single factor exhibited values ranging from 0.42 to 0.72. The results of the reliability test indicated that the internal consistency coefficient was 0.77, while the test–retest reliability coefficient was 0.99. The internal reliability coefficient, as shown by Cronbach’s Alpha, in this study was found as 0.82.

#### 2.3.3. Regulation of Emotions Questionnaire (REQ)

This measurement tool, which has been adapted to Turkish culture, was created by Philips and Power (2007). REQ aims to measure adolescents’ emotion regulation at the level of internal and external functioning. The exploratory and confirmatory factor analyses for validity showed that the scale consists of four factors, which are external functional, external dysfunctional, internal functional, and internal dysfunctional emotion regulation, and 18 items. This tool defines internal emotion regulation as the spontaneous regulation of emotions in the absence of external stimuli, whereas external emotion regulation is the regulation of emotions in response to external stimuli. “Functional” refers to purposeful functioning, while “dysfunctional” refers to situations that are ineffective for the purpose. Cronbach’s alpha reliability coefficients of the sub-dimensions of the scale were as follows: 0.74 for the sub-dimension of internal functional emotion regulation, 0.76 for the sub-dimension of extrinsic dysfunctional emotion regulation, 0.68 for the sub-dimension of internal dysfunctional emotion regulation, and 0.57 for the sub-dimension of extrinsic functional emotion regulation, respectively. The test–retest reliabilities were also found as 0.51, 0.70, 0.56, and 0.52, respectively (Duy & Yıldız, 2014). Higher scores mean a higher level of emotion regulation in the related sub-dimension. It is important to note that the total score of the scale is unattainable. Acknowledging the close relationship of irrational beliefs with negative thinking and emotions, this study uses 5 items each from the sub-dimensions of external and internal dysfunctional emotion regulation. The internal reliability coefficients were calculated; the one for the sub-scale of internal dysfunctional emotion regulation was found as 0.86 and that for the sub-scale of external dysfunctional emotion regulation was 0.76.

#### 2.3.4. Social Anxiety Scale for Adolescents (SAS-A)

This instrument, originally developed to assess social anxiety in children (La Greca et al., 1988), was subsequently adapted to adolescents, but the original 22-item structure was retained. Four items were excluded from the scoring, and a three-factor structure was identified: fear of negative evaluation (FNE), general social avoidance and distress (SAD-G), and restlessness, social avoidance, and distress in new situations (SAD-N). The internal consistency coefficients of the scale varied from 0.66 to 0.91 (La Greca & Lopez, 1998). The adaptation of the measurement tool to Turkish culture revealed that the 22 items with filler items and the 3-factor structure with the internal consistency coefficients of (FNE: 0.83), (SAD-G: 0.68) and (SAD-N: 0.71) and the correlation value of the social phobia scale for children and adolescents (r = 0.75) were appropriate for use. The two half reliability coefficients of this scale adapted to Turkish culture are between 0.67 and 0.85 (Aydın & Sütçü, 2007). The 5-point Likert-type scale does not include items with reverse scoring. Later, this study assessed the internal reliability of the instruments using Cronbach’s alpha coefficients. These coefficients were as follows: 0.87 for FNE, 0.76 for SAD-G, 0.79 for SAD-N, and 0.84 for the total score of the scale. These values were obtained through a reliability analysis conducted to evaluate the suitability of the instruments for use with 16–17-year-old high-school students.

### 2.4. Data Analysis

This research is designed in two stages. The first study involved the analyses required for scale development, while the second study focused on the analyses for the verification of the hypotheses of the model. This research employs SPSS 26.0 for descriptive statistics, correlational relationships, reliability coefficients and exploratory factor analysis based on construct validity, AMOS for confirmatory factor analysis and hypothesis testing in the mediation model, JAMOVI for McDonald’s ω, which is the internal reliability coefficient, and Microsoft Excel programmes to calculate validity-related average variance extracted (AVE) and composite reliability (CR) values.

In Study 1, KMO and Bartlett analysis was conducted on the data collected from 520 students (272 girls and 242 boys; age $\bar{X}$ = 15.2), once 23 incomplete data points were removed from the original 543 data points. This was completed prior to the onset of the construct validity assessment in the scale development phase. The initial step involved the execution of a Composite Reliability (CR) analysis, utilising the outcomes derived from the Exploratory Factor Analysis (EFA) to ascertain the convergent validity of PSAS. Next, the CR analysis was conducted based on the results of the exploratory factor analysis. To establish the construct validity, confirmatory factor analysis was performed using the maximum likelihood technique with the data collected from a total of 210 secondary and high school students (109 girls and 101 boys; age $\bar{X}$ = 14.9). Subsequently, due to the high correlation between social anxiety and public speaking anxiety (APA, 2013; Beidel & Turner, 2007), the SAS-A and PSAS were simultaneously administered to 160 students (82 girls, 78 boys; age $\bar{X}$ = 15.1) to assess and calculate the criterion validity, test–retest reliability, and internal consistency coefficient of the PSAS. Convergent validity level was determined using the average variance extracted (AVE) values, calculated considering the factor loadings (Fornell & Larcker, 1981), and the composite reliability (CR) values, calculated considering the EFA results.

Before testing the model (Figure 1) with the hypotheses in Study 2 through AMOS, a power analysis was carried out to ascertain whether the sample size was of sufficient size. This study applied Monte Carlo power analysis (Schoemann et al., 2017), which is an approach designed for multiple mediation models, to ascertain the minimum sample size required for the model to be tested. Assuming that there are moderate relationships between the variables in the model, the minimum sample size must reach 95% confidence; a power of 0.80 (Cohen, 2013) was calculated using 10,000 Monte Carlo replications with 20,000 drawings. The normality of the data was assessed using Skewness and Kurtosis coefficients. The values obtained ranged between 1.96 and −1.96, indicating that the data were normally distributed (Tabachnick & Fidell, 2019). Subsequently, Harman’s single factor test was employed to investigate the potential for common method bias in model-based studies. Consequently, all items in the scales used in this study were collected under a single factor without rotation, and exploratory factor analysis was performed. If the total variance explained is below 50%, the model cannot be tested due to the potential presence of common method bias (Fuller et al., 2016). The results of the test yielded that the maximum component total variance was 29.03%. This simply implies there is no common method bias for the mediation analysis in this study.

The Pearson’s Product Moment Correlation coefficient (r) was used to determine the relationships between the variables in this study. The bootstrapping (resampling) method was then employed to identify the mediating role of internal dysfunctional emotion regulation and external dysfunctional emotion regulation in the effect of irrational beliefs on public speaking anxiety in adolescent students.

## 3. Findings

### 3.1. Study 1. Validity and Reliability of the Public Speaking Anxiety Scale (PSAS)

Exploratory factor analysis, confirmatory factor analysis and criterion scale validity analyses were conducted to assess the validity of the PSAS. The reliability of the scale was tested through internal consistency and test–retest analysis.

#### 3.1.1. Findings on Validity

Exploratory factor analysis (EFA), composite reliability (CR) analysis, confirmatory factor analysis (CFA), criterion scale validity and average variance extracted (AVE) analysis were carried out to test the validity of the PSAS.

#### 3.1.2. Findings from the Exploratory Factor Analysis

**Figure 2:**
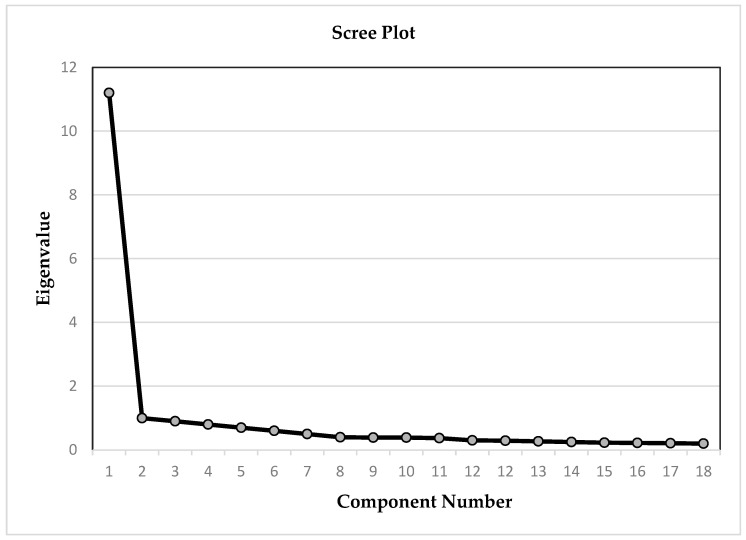
Eigenvalue factor graph of the PSAS.

To finalise the structure of the PSAS, exploratory factor analysis was conducted on the data obtained from a total of 520 adolescents. Prior to the exploratory factor analysis, KMO and Bartlett analyses were performed to determine the size of the sample. The KMO and Bartlett analyses were carried out to ascertain whether the requisite conditions for the factor analysis were met. The results of the analyses indicated that the data from 520 adolescents (272 girls and 248 boys; age $\bar{X}$ = 15.6) were sufficient for the factor analysis (KMO = 0.971 for PSAS). A KMO value between 0.80 and 1.0 is indicative of an adequate sample size (Tabachnick & Fidell, 2019). Bartlett’s Test of Sphericity was conducted to assess the multivariate normality of the data (χ2 = 9038,112; df = 406; *p* = 0.000) and this revealed that the assumption was met (*p* < 0.001). Next, exploratory factor analysis was applied to the PSAS, comprising 29 items. The first step of the exploratory factor analysis involved principal component analysis. As there was one eigenvalue above 1, it was concluded that the scale had a single-factor structure supported by the slope accumulation and scree-plot graphs (Figure 2). As the scale would have a single factor structure, no rotation technique was applied. Later, items with item loadings below 0.45 were removed. Lastly, a panel of 10 experts assessed the content validity of the resulting 18 items. Table 1 presents the structure and other psychometric data on validity obtained in the factor analysis.

As illustrated in Table 1, the results of the exploratory factor analysis indicated that the factor loadings range from 0.517 to 0.767 within the resulting single-factor 18-item structure. The eigenvalue of the PSAS in one factor is 11.686, with 18 items explaining 64.922% of the variance. The results further demonstrated that the items exhibit a relatively homogeneous structure. In the next step, confirmatory factor analysis was conducted to validate the single factor structure identified in the exploratory factor analysis.

#### 3.1.3. Findings from the Confirmatory Factor Analysis

**Figure 3:**
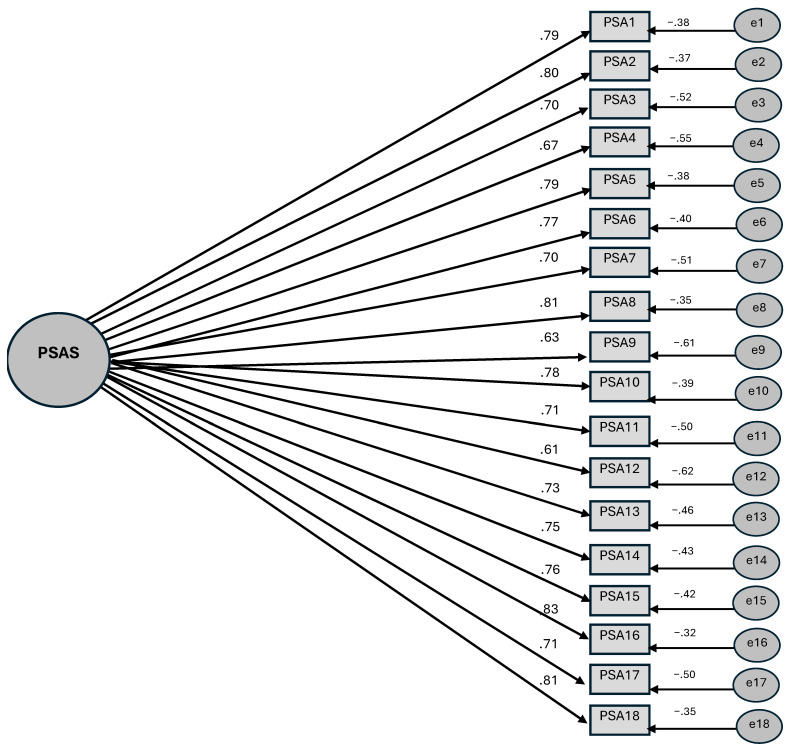
Confirmatory factor analysis of the PSAS—standardised path coefficients.

To perform confirmatory factor analysis, this study drew on the data obtained from the sample with a different yet closely related age group compared to that used in the exploratory factor analysis. To perform confirmatory factor analysis, data were collected from a total of 210 students (109 girls, 101 boys; age $\bar{X}$ = 14.9). Figure 3 presents the factor loadings in the confirmatory factor analysis.

The results of the confirmatory factor analysis in Figure 3 indicates that the goodness-of-fit indices for the PSAS are as follows: χ2/df = 2.453, *p* < 0.001, RMSEA = 0.092, GFI = 0.97, AGFI = 0.94, CFI = 0.99, NNFI = 0.96 and IFI = 0.91. Thus, the fit indices of the PSAS are within the acceptable range (Hair et al., 2013). However, it was observed that the RMSEA value did not correspond to the acceptance value stipulated by Hair et al. (2013). Consequently, an improvement modification was implemented between the two items, as the modification index between item 3 (I sweat before I start speaking in public) and item 4 (I experience hot or cold flushes before I start speaking in public) in the final form of the scale exhibited the highest value. Following the conclusion of the modification process, it was determined that the RMSEA value was 0.075, and the confidence intervals (lower limit of 90% confidence interval [LO-90] = 0.068, upper limit of 90% confidence interval [HI-90] = 0.082) were found to be within the acceptable range. Additionally, the SRMR value was recorded as 0.0309, and the previous values were also found to be within an acceptable range.

#### 3.1.4. Findings on Validity in Criterion Scale, Average Variance Extracted (AVE) and Composite Reliability (CR) Analysis

To test the criterion scale (conformity) validity of the PSAS, the 22-item SAS-A, four of which are filler items, was adapted to Turkish culture by Aydın and Sütçü (2007). The results of the data collected from 160 students (82 girls, 78 boys; age $\bar{X}$ = 15.1) indicated a significant correlation between the total score of the PSAS and the total scores of the SAS-A, with a Pearson’s product moment correlation coefficient (r = 0.87, *p* < 0.01). Therefore, the PSAS demonstrates the criterion scale validity. Further, to test the convergent validity, average variance extracted (AVE) analysis was conducted using Microsoft Excel, and the result was 0.55. After the EFA and before the CFA, the composite reliability (CR) (0.95) was calculated using Microsoft Excel. This result and the AVE result were evaluated together. Considering that the AVE value above 0.50 and CR value above 0.70 imply convergent validity (Hair et al., 2013; Fornell & Larcker, 1981), the PSAS has an established convergent validity.

#### 3.1.5. Findings on Reliability

The reliability of the PSAS was tested using internal consistency coefficients and test–retest results. The findings are presented based on the data obtained from a total of 210 students (109 girls, 101 boys; age $\bar{X}$ = 14.9).

#### 3.1.6. Internal Consistency Findings

The reliability of the scale was tested using Cronbach’s alpha, split-half and McDonald’s omega-ω reliability coefficient values. Table 2 offers the results of the reliability analysis of the PSAS.

Table 2 shows the internal consistency coefficients of the PSAS: Cronbach’s alpha, 0.957; Guttman split-half, 0.964; and McDonald’s omega-ω, 0.957. These results simply mean that the 18-item form of the PSAS has high internal consistency coefficients; therefore, this scale can yield consistent measurements.

### 3.2. Repeated Test Findings

The PSAS was administered to 160 students at three-week intervals to determine the reliability of the repeated tests. The Pearson correlation coefficient between the two measurements obtained was calculated as r = 0.84, which is significant at the *p* < 0.01 level. These results demonstrate that the repeated measurements of the PSAS are consistent.

### 3.3. Study 2: Findings on the Correlational and Mediating Effects of Internal Dysfunctional and Extrinsic Dysfunctional Emotion Regulation on Public Speaking Anxiety and Irrational Beliefs in High School Students

As demonstrated in Table 3, a positive and significant relationship was obtained between speaking anxiety and irrational beliefs (r = 0.42, *p* < 0.05), internal dysfunctional emotion regulation (r = 0.46, *p* < 0.05), and external dysfunctional emotion regulation (0.19, *p* < 0.05). The findings of this study demonstrated a positive and significant relationship between irrational beliefs and internal dysfunctional emotion regulation (r = 0.51, *p* < 0.05), as well as a low but significant relationship between external dysfunctional emotion regulation (r = 0.07, *p* > 0.05). A significant positive relationship was finally obtained between internal dysfunctional emotion regulation and external dysfunctional emotion regulation (r = 0.28, *p* < 0.05). Upon analysis of the descriptive findings related to the variables, it is evident that the reliability (Cronbach’s alpha) of the measurement tools is high. As demonstrated in the above table, it can be concluded that the data follow a normal distribution, since the skewness/kurtosis coefficients for each measurement tool are in the normal range. Moreover, it has been established that the anticipated values for the absence of multicollinearity in mediation analysis are within the range of Tolerance > 20 and VIF < 10 (Field, 2009).

**Figure 4:**
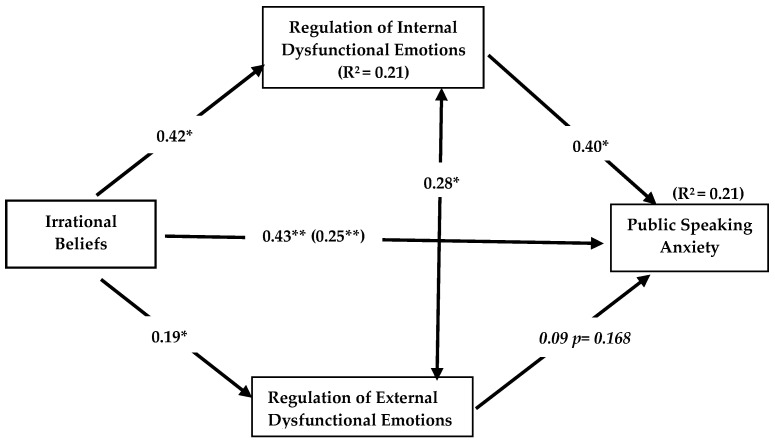
Mediating roles of internal dysfunctional emotion regulation and external dysfunctional emotion regulation in the effect of irrational beliefs on public speaking anxiety. ** *p* < 0.01, * *p* > 0.05.

Figure 4 includes the findings regarding the mediating roles of internal and external dysfunctional emotion regulation in the effect of irrational beliefs on speaking anxiety.

As illustrated in Figure 4, the overall impact of irrational beliefs on speaking anxiety was found to be statistically significant (β = 0.43, 95% CI [0.32, 0.54], *p* < 0.01). On the other hand, while the direct effect of irrational beliefs on internal dysfunctional emotion regulation is positively significant (β = 0.42, 95% CI [0.52, 0.36], *p* < 0.01), the effect of internal dysfunctional emotion regulation on speaking anxiety is also positively significant (β = 0.40, 95% CI [0.30, 0.52], *p* < 0.01). The direct effect of irrational beliefs on speaking anxiety was also found to be significantly positive (β = 0.25, 95% CI [0.22, 0.42], *p* < 0.01). The data indicated that irrational beliefs exhibit an indirect effect on speaking anxiety through internal dysfunctional emotion regulation (β = 0.17, 95% CI [0.13, 0.21], *p* < 0.01). Irrational beliefs explained 0.31 of the total variance of speaking anxiety through internal dysfunctional emotion regulation (R^2^ = 0.31). Therefore, it is reasonable to conclude that both the direct effect of irrational beliefs on speaking anxiety and the indirect effect of internal dysfunctional emotion regulation on speaking anxiety are significant. Moreover, this study suggests that internal dysfunctional emotion regulation plays a partial mediating role in the effect of irrational beliefs on speaking anxiety.

However, the direct effect of irrational beliefs on the regulation of external dysfunctional emotions was found to be significantly positive (β = 0.19, 95% CI [0.32, 0.06], *p* < 0.01; R^2^ = 0.08). Yet, the effect of regulation of external dysfunctional emotions on speaking anxiety was found to be insignificant, despite being in a positive direction (β = 0.09, *p* > 0.01). Therefore, there is no discernible mediating role of external dysfunctional emotion regulation in the effect of irrational beliefs on speaking anxiety, as the condition that the effect of the mediating variable on the dependent variable should be significant is not met.

Lastly, the direct effect of internal dysfunctional emotion regulation on external dysfunctional emotion regulation was found to be significantly positive (β = 0.28, 95% CI [0.09, 0.39], *p* < 0.01). Table 4 presents the path coefficients and bootstrapping confidence intervals for the total, direct and indirect effects among the variables.

Table 4 shows that the effect of irrational beliefs on public speaking anxiety is significant in total, directly and indirectly through internal dysfunctional emotion regulation. Moreover, when extrinsic dysfunctional emotion regulation assumed a mediating role in the effect of irrational beliefs on public speaking anxiety, this was not significant enough to impact the direct effect. Thus, dysfunctional emotion regulation seems to play a mediating role in the effect of irrational beliefs on public speaking anxiety. This study can suggest that external dysfunctional emotion regulation does not have a mediating role in the effect of irrational beliefs on public speaking anxiety.

## 4. Discussion

The first stage of this two-stage study presented the validity and reliability results of the PSAS. The results indicated that the PSAS with its 18 items and 1 dimension is a valid instrument for adolescents. The second stage examined the mediating role of internal dysfunctional and external dysfunctional emotion regulation in the effect of irrational beliefs on public speaking anxiety in adolescents. To test the hypotheses, this study analysed the relationships between variables and ascertained that the relationship and effect of external dysfunctional emotion regulation on public speaking anxiety was not significant. However, other variables had significant relationships with each other. Thus, this study concluded that internal dysfunctional emotion regulation plays a mediating role in the effect of irrational beliefs on public speaking anxiety. It is also important to note here that external dysfunctional emotion regulation did not have a mediating role in the effect of irrational beliefs on public speaking anxiety.

The validity and reliability of the PSAS is measured with a sample of secondary school students aged 12–17. As mentioned earlier, the non-Turkish measurement tools on public speaking anxiety were developed by psychology or behavioural sciences researchers (Bartholomay & Houlihan, 2016) for university students. It is notable that many scales in Turkey are developed and adapted only by educators of language and are aimed at university students (Çabuker et al., 2020; Demir & Melanlıoğlu, 2014; Gündüz & Demir, 2021; Keşaplı & Çifçi, 2019; Kinay & Özkan, 2014; Sevim, 2012; Yaman & Sofu, 2014). In consideration of the substantial correlation between public speaking anxiety and social anxiety (APA, 2013) and the recognised prevalence of social anxiety in adolescence as previously outlined, it is anticipated that the PSAS, developed by researchers specialising in psychological counselling and language education with a focus on adolescents, will make a pioneering contribution to the existing literature.

As previously stated, social anxiety and related public speaking anxiety have been demonstrated to have a detrimental effect on adolescents’ social relationships, daily life skills, and academic performance. It is posited that the identification of students experiencing such anxiety through PSAS will have a preventive effect on their course success, daily life, and social relationships in school guidance-based studies. On the other hand, Gallego et al. (2022) asserted that self-report is a more efficacious method of measuring speaking anxiety than the results obtained by observing physiological reactions and the audience. Furthermore, McCroskey (1982) posited that self-report results on public speaking anxiety are more reliable than physiological arousal indicators and observer evaluations. In this context, it is hypothesised that the single-factor PSAS will serve as an effective measurement tool for adolescents.

Based on the results, this study supports the hypothesis that the direct effect of irrational beliefs on public speaking anxiety in adolescents is positively significant. Individuals with irrational beliefs believe that they will be criticised by others in situations requiring public performance, because they make negative self-evaluations (Blöte et al., 2014; Chiu et al., 2021; Leigh & Clark, 2016). Irrational beliefs, such as negative evaluation (David et al., 2009), lead to high levels of social anxiety (Li et al., 2019). The results of this study are congruent with the previous research (Davison & Zighelboim, 1987; Şahan & Kahtali, 2021; Tovilović, 2004) and the relevant literature (Lohr & Bonge, 1981) indicating that social anxiety and public speaking anxiety have a significant relationship with irrational beliefs. Here, it should be noted that experimental and descriptive studies in the literature were conducted with university students. For that reason, the results of this current study are pioneering in the literature, particularly because it reveals the relationship between irrational beliefs and speaking anxiety in adolescents.

This study also validated the hypotheses that the direct effect of irrational beliefs on internal and external dysfunctional emotion regulation is significant. The close relationship between people’s beliefs and the emotions that are the result of those beliefs (Ellis, 2013) is well established. David et al. (2009) stated that the faulty cognitive processes of people with irrational beliefs negatively affect their emotions. This is observed particularly in adolescents (Bernard, 1984). Adolescents experience negative emotions due to irrational beliefs and may rely on dysfunctional ways of regulating these negative emotions (Rice et al., 2017). Irrational beliefs, which convey negativity, can cause many psychological consequences such as increased stress level specific to adolescence (Yıldız et al., 2018), expression of anger and aggressive behaviour (Fives et al., 2011), and decreased life satisfaction (Çivitçi, 2009). When one experiences such problems, their irrational beliefs can lead to negative regulation of emotions through dysfunctional cognitive processes (Ellis, 2013). Individuals can have more positive emotions when they regulate their emotions in a functional way by changing irrational beliefs (David et al., 2009; Ellis, 2013). Predatu et al. (2020) posited that irrational beliefs play a significant role in the regulation of an individuals’ emotions. Those who misinterpret beliefs about the causes of their emotions experience difficulties in regulating their emotions. Hong and Kangas’ (2022) meta-analysis of 25 studies on emotion regulation and beliefs found a positive relationship between positive belief in the controllability of emotions and positive emotion regulation. Moreover, Vacca et al. (2023) have posited that emotional and cognitive regulation play a substantial role in the depression and anxiety exhibited by adolescents subjected to bullying. In summary, the evidence suggests that a regulatory approach targeting cognitions and emotions is an effective strategy to reduce anxiety in adolescents at risk. The present study’s findings, in congruence with extant research, demonstrate a positive correlation between irrational beliefs and the dysfunctional regulation of emotions.

The hypothesis that internal and external dysfunctional emotion regulation has a direct effect on public speaking anxiety was partially validated. Although internal dysfunctional emotion regulation had a direct effect on public speaking anxiety, external dysfunctional emotion regulation showed no direct effect. The present study’s findings are corroborated by the assertions of Leigh and Clark (2016), who hypothesised that the emotional responses to social anxiety are attributable to impaired or dysfunctional self-regulation. Niles and Craske (2019) reported that lacking emotion regulation processes cause public speaking anxiety, and that the emotions of those experiencing such anxiety originate internally and they cannot be understood from an external perspective. Hannesdóttir (2007) found that internally organising positive emotions and focusing on them was effective in reducing levels of public speaking anxiety. It is also known that emotion dysregulation that causes anxiety is generally caused by person-specific cognitive processes (Hannesdóttir & Ollendick, 2007). In other words, dysfunctional emotion regulation strategies that result from internally based cognitive regulations cause anxiety. Jazaieri et al. (2015) stated that the failure in emotion regulation processes such as attention, evaluation, and taking responsibility towards oneself results in social anxiety. On the other hand, the absence of correlation between external, dysfunctional emotion regulation, such as anger and aggression, and internal evaluations is hypothesised to be a contributing factor to this outcome. Previous research (Brühl et al., 2013) suggests that dysfunctional emotion regulation, which brings about social anxiety, is caused by problems with neurons rather than external factors. As can be seen, numerous studies indicated a positive relationship between dysfunctional emotional regulation and speaking anxiety and social anxiety. In light of these, it is reasonable to argue that internal dysfunctional emotion regulation in particular has an impact on social anxiety and speaking anxiety. Considering the results of the research in the literature and the results of this study, one may claim that more internal dysfunctional emotion regulation has a direct effect on public speaking anxiety.

Lastly, this study validated the hypothesis that the indirect effect of irrational beliefs on public speaking anxiety through external dysfunctional emotion regulation in adolescent students is positively significant. On the other hand, this study refutes the hypothesis that the indirect effect of irrational beliefs on public speaking anxiety through external dysfunctional emotion regulation in adolescent students is positively significant. This suggests that only internal dysfunctional emotion regulation mediated the effect of irrational beliefs on public speaking anxiety in adolescent students. Previous research also reported a positive effect of irrational beliefs on speaking anxiety, as mentioned above. Cécillon et al. (2024) posited that individuals must be capable of regulating their metacognitive systems and emotions in order to experience trait anxiety. Urfa and Aşçı (2023) showed that negative emotions play a mediating role in the effect of irrational beliefs on performance. The results of the previous study seem to support the results of this study if public speaking is also considered as a performance. Irrational beliefs negatively affect the functional regulation of emotions due to faulty cognitive processes (Ellis, 2013). In a similar vein, Sackl-Pammer et al. (2019) posited that emotion dysregulation based on cognitive errors is efficacious in adolescents with social anxiety. In this context, the finding that rumination, self-blame and fear of making mistakes—all of which are characteristics of internal dysfunctional emotion regulation—are also examples of irrational beliefs indicates that public speaking due to social anxiety has a joint effect on anxiety. Therefore, dysfunctional regulation of emotions in activities such as public speaking is believed to cause anxiety. For that reason, cognitive errors and misinterpretations can be influential not only in the process of processing information, but also in the process of making sense of and regulating emotions. Given the fact that social anxiety is driven by negative subjective interpretations and evaluations (Lin et al., 2021), it is reasonable to claim that internal dysfunctional emotion regulation plays a mediating role in the effect of irrational beliefs on public speaking anxiety.

## 5. Limitations and Future Research Recommendations

The present study is not without its own limitations. In light of the aforementioned limitations and the results obtained in the research, the suggestions are as follows:

(1) The high degree of correlation between the PSAS and the criterion scale SAS-A may have been partially affected by the common method variance. In subsequent studies, a range of methods or measurement tools may be employed to ascertain more substantial relationships in the context of criterion scale validity.
(2) Despite the multifaceted nature of public speaking anxiety, manifesting in behavioural, physiological and cognitive domains, the PSAS utilised in this study encompasses only a single dimension. In order to facilitate the formation of these dimensions with greater clarity, it is recommended that the items be elevated to a more distinct level through the implementation of multiple pilot applications during the scale development process.
(3) The findings of the present study were only obtained from data collected from adolescents in Turkey. The question of the PSAS’s validity in other cultures can be investigated.
(4) This study was conducted on cross-sectional data. It is posited that a longitudinal study of the same model in a process such as adolescence, where variability is experienced to a greater extent, will engender more generalisable results.
(5) This study overlooked the issue of gender-related measurement invariance with regard to a significant variable in adolescence, namely public speaking anxiety. In subsequent studies, the measurement invariance or level of gender-related PSAS can be determined.
(6) It has previously been demonstrated that internal dysfunctional emotion regulation strategies are rooted in the cognitive process (Carthy et al., 2010; Dixon et al., 2020). In consideration of the mediating role identified in this study, it is posited that school-based group activities for emotion regulation will be efficacious in preventing adolescents from experiencing social anxiety and similarly public speaking anxiety.

## 6. Conclusions

In conclusion, the Public Speaking Anxiety Scale developed in this study exhibits reliability in measuring the intended constructs in adolescents. This study further concludes that adolescents’ irrational beliefs shape their public speaking anxiety through internal dysfunctional emotional regulation, which is a finding supported by the respective literature and other similar research. A final noteworthy finding of this study is that external dysfunctional emotion regulation did not mediate the effect of irrational beliefs on public speaking anxiety.

## Figures and Tables

**Table 1:** Findings of exploratory factor analysis on the PSAS.

Last Item No	First Item No	Factor Loadings	Item Total Test Correlation
1	2	0.610	0.758
2	5	0.640	0.774
3	7	0.665	0.698
4	8	0.643	0.682
5	9	0.599	0.774
6	11	0.732	0.750
7	14	0.689	0.678
8	16	0.664	0.779
9	17	0.599	0.626
10	18	0.767	0.776
11	20	0.510	0.702
12	21	0.696	0.601
13	22	0.612	0.713
14	23	0.614	0.725
15	24	0.644	0.733
16	27	0.722	0.803
17	28	0.517	0.704
18	29	0.683	0.795
Eigenvalue		11.686	
Variance explained		64.922	

**Table 2:** Internal consistency levels.

	Number of Items	Cronbach’s Alpha	Guttman Split-Half	McDonald’s Omega-ω
PSAS	18	0.957	0.964	0.957

**Table 3:** Descriptive findings, correlation coefficients (r), and multicollinearity values.

Variables	1	2	3	4	Tol	VIF
1-Public speaking anxiety	1					
2-Irrational beliefs	0.42 **	1			0.783	1.277
3-Regulation of internal dysfunctional emotions	0.46 **	0.51 **	1		0.748	1.336
4-Regulation of external dysfunctional emotions	0.19 **	0.07 *	0.28 **	1	0.916	1.092
α	0.96	0.82	0.86	0.76		
Skewness	0.242	−0.219	0.181	1.341		
Kurtosis	−0.926	−0.424	−0.607	1.936		
Average	54.73	55.99	13.70	10.01		
Sd.	20.72	10.82	4.59	4.25		

** *p* < 0.01, * *p* > 0.05.

**Table 4:** Total, direct and indirect effects of irrational beliefs on speaking anxiety and bootstrapping confidence intervals in adolescents.

Types			Total Effect (% 95 CI)	Direct Effects (% 95 CI)	Indirect Effect (% 95 CI)	Decision
Irrational Beliefs	→	Public Speaking Anxiety	−0.43 ** (0.32 **, 0.54 **)	0.25 ** (0.22 **, 0.42 *)	−0.17 ** (0.13 **, 0.21 **)	Partial Mediation
Irrational Beliefs	→	Regulation of Internal Dysfunctional Emotions		0.42 ** (0.52 **, 0.36 **)		
Regulation of Internal Dysfunctional Emotions	→	Public Speaking Anxiety		0.40 ** (0.30 **, 0.52 **)		
Irrational Beliefs	→	Regulation of External Dysfunctional Emotions		0.19 ** (0.32 **, 0.06 **)		No intermediation was observed.
Regulation of External Dysfunctional Emotions	→	Irrational Beliefs		0.09 (*p*; 0.168 > 0.01)		

** *p* < 0.01; * *p* > 0.05.

## Data Availability

Data are contained within the article.
